# Intrahost viral evolution of SARS-CoV-2 infections in rheumatic versus hematological patients with severe iatrogenic immunosuppression

**DOI:** 10.3389/fmed.2026.1715096

**Published:** 2026-04-30

**Authors:** Emmanouil Karofylakis, Theodoros Loupis, Andromachi Blizou, Eleni Ntalaouti, Eirini Maria Stergioti, Giannis Vatsellas, Sotirios Tsiodras, Anastasia Antoniadou, Aggelos Banos, Konstantinos Thomas

**Affiliations:** 1 4th Department of Internal Medicine, National and Kapodistrian University of Athens School of Medicine, Attikon University General Hospital, Chaidari, Greece; 2 Hematology Research Lab, Clinical, Experimental and Translational Research Center, Biomedical Research Foundation Academy of Athens, Athens, Greece; 3 Laboratory of Autoimmunity and Inflammation, Center of Clinical, Experimental Surgery and Translational Research, Biomedical Research Foundation Academy of Athens, Athens, Greece; 4 Greek Genome Center, Biomedical Research Foundation Academy of Athens, Athens, Greece

**Keywords:** immunosuppression, intrahost evolution, rituximab, SARS-CoV-2, virome sequencing

## Abstract

**Objectives:**

Immunocompromised patients with B-cell depletion are at risk of ongoing SARS-CoV-2 replication that can lead to intra-host genomic divergence, immune escape during treatment and potentially emergence of novel variants.

**Methods:**

We conducted a prospective observational study including severely immunocompromised rheumatic and hematologic patients with laboratory-confirmed COVID-19. SARS-CoV-2 virome sequencing was performed on respiratory samples, followed by phylogenetic and intrahost single nucleotide variants (iSNVs) analysis. In a subset of patients, lymphocyte subpopulations were assessed by flow cytometry.

**Results:**

Twenty-six patients were included (median age: 71 years, rheumatic disease: 50%, treated with B-cell depleting agent: 88%, prior COVID-19 infection: 68%, relapsing COVID-19: 65%, ≥ 3 vaccine doses: 68%). Twenty-two patients (85%) were hospitalized with 23% mortality. Three unique RdRP and 3CLPro gene mutations were identified with a prevalence < 0.1%, not known to confer antiviral resistance. We identified 30 mutations in the spike gene with prevalence < 0.1% or leading to a new N-glycosylation site. In patients with paired samples, we noticed a statistically significant iSNVs accumulation with an average substitution rate per site per day of 7 × 10^–6^ (*p* < 0.001). We did not observe statistically significant difference in the substitution rates between rheumatic and hematologic patients.

**Conclusion:**

In our cohort, a significant proportion of patients had relapsing COVID-19. Unique and rare mutations were detected mainly in the spike gene, whereas those in the polymerase and protease genes were not of known significance. We found a positive correlation of iSNVs accumulation with infection duration, without difference in substitution rates between rheumatic and hematologic patients.

## Highlights

- B-cell depleted patients are reservoirs for prolonged SARS-CoV-2 replication, leading to unique intrahost mutations.
- A positive correlation exists between duration of infection and intrahost single nucleotide variants (iSNVs) accumulation.
- No difference was noted in substitution rates between rheumatic and hematologic patients.

## Introduction

In May 2023, the World Health Organization downgraded coronavirus disease 2019 (COVID-19) pandemic as a global emergency. In the general population, hybrid vaccine and post-infection immunity has transformed COVID-19 into a mild upper respiratory tract infection. Immunocompromised patients, however, remain at risk for deleterious outcomes (1).

Early in the pandemic, rheumatic disease and multiple sclerosis registries noted an association of anti-CD20 monoclonal antibodies (mAbs), like rituximab, with increased COVID-19 severity, intensive care unit (ICU) admission rates and increased mortality (2–5).

Patients with hematologic malignancies, especially those on anti-CD20 therapies, are similarly at increased risk for severe COVID-19. Lower vaccine immunogenicity due to B-cell depletion has been associated with worse COVID-19 outcomes (6–8). Nevertheless, B-cell depletion continues to confer an increased risk for breakthrough infections in fully vaccinated individuals (9).

A unique characteristic of COVID-19 in immunocompromised patients is the risk for relapsing or protracted disease (10, 11). B-cell depletion, particularly when coupled with T-cell deficiencies, has been associated with prolonged PCR positivity and delayed culture clearance (12). This ongoing viral replication can lead to intra-host genomic divergence and immune escape mutations (13, 14). Community-wide genomic studies highlight the role of persistent infections in immunocompromised hosts as a “mutation reservoir” (15). However, little is known about the differences in the mutational accumulation rate between rheumatic and hematological patients treated with B-cell depleting mAbs.

Aiming to further our understanding of those vulnerable populations, we conducted a prospective cohort study focusing on viral genomic evolution during their course.

## Materials and methods

### Patients

This was a prospective, observational study conducted between January 2023 and November 2023 in Attikon University Hospital in Athens, Greece. We included serial patients ≥ 18 years old, with laboratory-confirmed COVID-19 infection of any severity (classified according to NIAID classification) and ≥ 1 of the following criteria for immunosuppression: ≥ 1 dose of any anti-CD20 depleting mAb during the last 12 months for any indication; allogeneic hematopoietic stem cell transplantation; induction chemotherapy for hematologic malignancies during the last 3 months. Laboratory-confirmed SARS-CoV-2 infection was defined as positive nucleic acid amplification testing (NAAT) at the time of admission or during hospitalization. Relapsing disease was defined according to already proposed definitions (16). All patients provided written informed consent. The study was approved by Institutional Ethics Committee of Attikon University Hospital (ID: 487/3–9-2020).

### Procedures

In all patients fulfilling the inclusion criteria, data regarding demographics, comorbidities, medications, history of prior COVID-19 infection or vaccination were collected. After the confirmation of SARS-CoV-2 NAAT positivity, a nasopharyngeal (NP) sample was sent for virome sequencing. NAAT-positive bronchoalveolar lavage (BAL) samples of eligible patients that underwent bronchoscopy for diagnostic purposes were also sequenced. Follow-up NP samples were collected weekly, at the time of discharge and in cases of readmission irrespective of the elapsed time between discharge and readmission. In a subset of patients, 10 mL of whole blood was collected for B- and T-cell lymphocyte immunophenotyping, as described in Supplementary Table 1.

### SARS-CoV2 virome sequencing and genome assembly

SARS-CoV2 virome sequencing was performed with the QIAseq^®^ SARS-CoV-2 targeted whole viral genome library prep kit. In brief, 5 μL RNA extracted from samples was reverse transcribed into cDNA using random primers. Following cDNA synthesis, primer pools based on the ARTIC v3 design were used in a high-fidelity multiplex PCR reaction to prepare two pools of 400 bp amplicons. The two enriched pools per sample were then pooled into a single tube, purified and sequencing ready Illumina libraries were constructed with the QIAseq FX DNA Library preparation kit. Sequencing was performed in a Novaseq6000 (2 × 150 bp) with approximately 1 million Paired End reads allocated to each sample.

### Quality control of SARS-CoV-2 genome sequences

Quality control of the raw sequence data (.fastq) was performed using FastQC^1^ and adapters and low quality bases were trimmed using Fastp.^2^ Next, the reads were aligned to the reference Wuhan genome (MN908947v3) using minimap2. ARTIC primers were removed using the ivar trim (iVar version 1.3, github.com/andersen-lab/ivar) setting parameters -e -m 30 q 20. The consensus genome was extracted in fasta format from the sorted BAM files and positions with coverage lower than 10 were replaced with an “N.” Resulted fasta files were uploaded to NextClade^3^ to filter out low quality sequences from the study. Lineage assignment was performed using the Phylogenetic Assignment of Named Global Outbreak Lineages (PANGOLIN) webtool (version 4.3). NextClade^4^ web application was used to assign clades, as well as to identify mutations (amino acid replacement, deletions etc.).

### Phylogenetic analysis

For the phylogenetic analysis, 500 randomly selected Greek SARS-CoV-2 sequences submitted between 01/01/2023 and 31/12/2023 from GISAID (17) were used (EPI_SET_241018zx, 10.55876/gis8.241018zx), along with 33 samples from our study. Multiple sequence alignment of these sequences with the SARS-CoV-2 reference genome (MN908947v3) was performed using MAFFT (v7.475) (18). Phylogenetic trees were constructed using IQ-TREE (v2.0.3) with the GTR model (19). Tree refinement (–date-confidence, –clock-rate 0.0008, –clock-std-dev 0.0002, –timetree, –root oldest) and visualization were performed using Augur (v25.2.0) and Auspice, respectively.^5^

### Structural visualization

Structural visualization of the SARS-CoV-2 spike glycoprotein was performed using the prefusion structure (PDB ID: 6XR8) in PyMOL (The PyMOL Molecular Graphics System, Version 3.0, Schrödinger, LLC). Mutation annotations per patient cohort were obtained with the CoVsurver tool (17) and mapped onto the structure for visualization (20).

### Intrahost single nucleotide variants (iSNVs) analysis

Intrahost single nucleotide variants (iSNVs) were called using iVar. Samples that had poor genome coverage (minimum 30x coverage for at least 75% of their genome) were excluded. The following criteria were followed for the resulting iSNVs: Variant frequency 0.1–0.8, variants above 0.8 were considered primary variants of the patients’ strain, while variants below 0.1 were excluded to avoid false positive calls or contaminants; *p* < 1 × 10^–3^; minimum depth of 30x; flagged from iVar as PASS. Variants in Ambiguous and homoplastic sites were excluded based on the UCSC recommendations (21). Indels were called but excluded from further analysis. For the timepoint comparison of longitudinal samples only the common genomic regions that had at least 30x coverage were kept.

### Statistical analysis

Continuous variables are presented as mean [ ± standard deviation (SD)] or median values [interquartile range (IQR)] and dichotomous variables are presented as absolute numbers (n) and frequencies (%). For the timepoint comparison, normality of the mutation count data was assessed and paired Student’s *t*-test was performed. Correlation analysis was conducted using Pearson’s method to evaluate the relationship between iSNV accumulation and time as both variables were continuous and met assumptions of linearity. Linear regression was applied to visualize iSNV accumulation over time as well as to calculate the substitution rate per site per day (slope-of-regression/30,000 bp). To evaluate differences in mutation accumulation between the rheumatic diseases (RA) and hematologic malignancies (HEM) subgroups, a fitted linear model (iSNVs ∼ Days × Diagnosis) was used, where the interaction term (Days × Diagnosis) tested whether mutation accumulation rates (slopes) differed between subgroups. In addition, normalized iSNV density (substitutions per base pair) was calculated on a per-gene basis both within cohorts and across all samples combined, in order to identify genes with the highest overall mutation density. To minimize bias introduced by longitudinal sampling, we restricted the analysis to unique iSNVs per patient, thereby preventing repeated detection of the same variant from inflating density estimates.

## Results

### Patient characteristics and outcomes

Twenty-six patients were included and equally distributed between systemic rheumatic diseases (RD) and hematologic malignancies (HEM). RD subgroup included patients with antineutrophil cytoplasmic antibodies-associated vasculitis (AAV, *n* = 5), systemic lupus erythematosus (SLE, *n* = 3), rheumatoid arthritis (RA, *n* = 2), retroperitoneal fibrosis (*n* = 1), central nervous system vasculitis (*n* = 1) and dermatomyositis (*n* = 1). HEM subgroup included patients with lymphoma (*n* = 9), chronic lymphocytic leukemia (*n* = 2), acute myeloid leukemia (*n* = 1) and multiple myeloma (*n* = 1). Twenty-three patients (88%) had a treatment history with a B cell depleting agent, mainly rituximab (*n* = 20) (Table 1).

**TABLE 1:** Patients’ descriptives.

Variable	Value
Male, n (%)	14 (54)
Age, years, median (IQR)	71 (54–77)
Diagnosis, n (%)	
Hematological malignancy	13 (50)
Systemic rheumatic disease	13 (50)
Prior COVID-19, n (%)	18 (69)
Number of SARS-CoV-2 vaccine doses, n (%)	
0	4 (15)
1	1 (4)
2	3 (12)
3	12 (46)
≥4	6 (23)
B-cell depleting therapies	23 (88)
Need for hospitalization, n (%)	22 (85)
Outcome, n (%)	
Discharge	20 (77)
Death from any cause within hospital	6 (23)

IQR, interquartile range; COVID-19, coronavirus disease 2019; SARS-CoV-2, Severe acute respiratory syndrome coronavirus 2.

Eighteen patients (68%) had a history of prior COVID-19 infection. In total, 68% of our cohort (18/26) had received ≥ 3 vaccine doses, while four have not received any vaccine. Twenty-two patients (85%) were hospitalized with a mortality rate of 23% (6/26). Sixty-five percent (17/26) of patients presented during an episode of clinical relapse in the context of protracted COVID-19.

Compared to HEM subgroup, RD patients were more likely to be women (61.5% vs. 31%, *p* = 0.12) and to be younger (60.4 ± 24.1 vs. 67.8 ± 10.9 years, *p* = 0.32), although the differences were not statistically significant. The subgroups did not differ in terms of prior COVID-19 history (69% vs. 69%, *p* = 1.0) and the number of vaccines (3 vs. 3, *p* = 0.16), neither. A significant difference was noted in outcome, since all deaths (*n* = 6) occurred in the HEM group (46% vs. 0%, *p* = 0.005).

Of 42 initially collected samples, 31 were included in the final virologic analysis with the rest being excluded due to low sequencing quality. A repeat second sample was obtained from eight patients at a median of 14 days. In one case, a third sample was obtained (12, 26 and 39 days after symptoms onset) (Supplementary Table 2).

Peripheral blood Immunophenotyping of 21 patients at baseline highlighted a B-cell depletion, while CD4^+^ and CD8^+^ T cell subpopulations remained relatively preserved (Supplementary Figure 1).

### Rare mutations in the pharmacologic targets of antivirals (Pro, Pol) and neutralizing antibodies (spike).

We studied the frequency of mutations of interest in our cohort comparing our data with two web-based public databases (Coronavirus Antiviral and Resistance Database, Stanford University and CoVsurver mutation analysis, GISAID). More specifically, we aimed to identify rare mutations in the genes encoding the RNA-dependent RNA polymerase (RdRP), the protease (3CLPro) and the spike protein, as these are the main pharmacologic targets of the currently available antiviral treatments. We identified three unique mutations from different patient samples both in the RdRP and the 3CLPro gene with a prevalence below 0.1% in these public databases. A literature search did not identify these mutations as potential risk factors for resistance to remdesivir, molnupiravir and nirmatrelvir/ritonavir, respectively. Regarding the spike gene, our comparative analysis identified 30 mutations with a prevalence below 0.1% or leading to a new potential N-glycosylation site (Table 2). The structural representation of the SARS-CoV-2 spike protein along with the amino-acid changes in positions known to be associated with altered host-cell receptor interaction (i.e., epitopes of monoclonal antibodies binding) or antigenicity is presented in Supplementary Figures 2A,B.

**TABLE 2:** List of selected mutations of interest in Spike (based on rarity or their effect on glycosylation sites), RNA-dependent RNA polymerase and viral protease.

Gene of interest	AA change	Stanford University Coronavirus antiviral & resistance database^†^ prevalence (%)	GISAID CoVsurver mutation analysis^‡^prevalence (%)
RdRP			
	D63N	< 0.01	0.11
	A449V	–	0.05
	I536V	–	0.01
3CLPro			
	S62A	< 0.01	0.00 (Occurred 1 time)
	A234V	–	0.05
	P241L	–	0.06
Spike			
	P9S	–	0.01
	Q14H	–	0.05
	Q14R	< 0.01	0.01
	T29I	–	0.09
	R78S	–	0.02
	H146K	< 0.01	0.06
	Y170H	–	0.01
	K182I	< 0.01	0.01
	L229F	–	0.01
	H245Y	–	0.07
	G257D	–	0.06
	P330L	< 0.01	0.01
	P330S	–	0.02
	A475V	0.03	0.04
	N481S	< 0.01	0.00 (Occurred 14 times)
	G504S	< 0.01	0.00 (Occurred 25 times)
	P521S	< 0.01	0.09
	P521T	< 0.01	0.01
	K558T*	< 0.01	0.00 (Occurred 2 times)
	A647V	< 0.01	0.00 (Occurred 40 times)
	A653V	–	0.09
	Y660H	< 0.01	0.00 (Occurred 40 times)
	D839V	< 0.01	0.01
	A845V	–	0.03
	A890V	–	0.01
	L922F	–	0.02
	A958D	< 0.01	0.00 (Occurred 4 times)
	I1179T	< 0.01	0.00 (Occurred 1 time)
	P1263Q	< 0.01	0.04

3CL^Pro:^ 3C-like protease, RdRP: RNA-dependent RNA polymerase.

† https://covdb.stanford.edu/ (accessed at 05/10/2024).

‡ https://gisaid.org/database-features/covsurver-mutations-app/ (accessed at 05/10/2024).

*Mutation creates a new potential N-glycosylation site.

**FIGURE 1:**
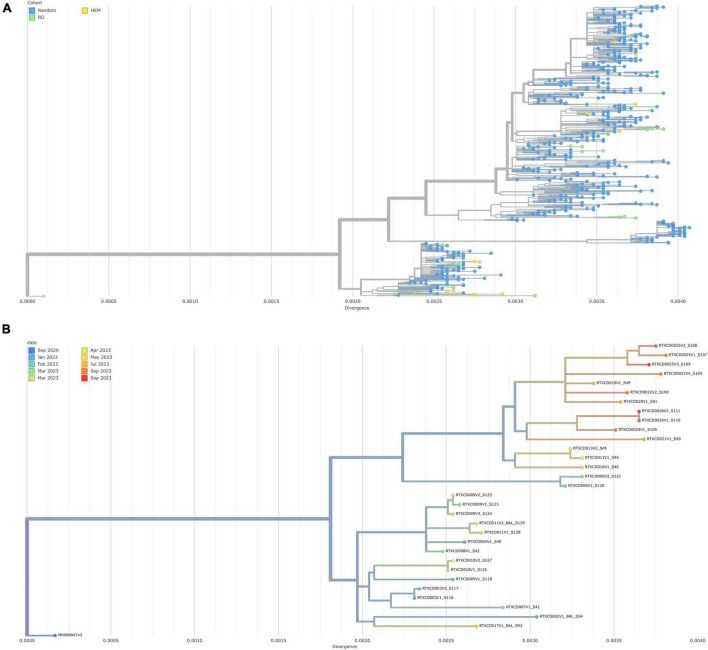
**(A)** Phylogenetic tree of patients’ samples with 500 samples in the background by divergence on x-axis. **(B)** Phylogenetic tree of patients’ samples only by divergence on x-axis.

In a zoomed-out capture of the virome compositions, phylogenetic trees reveal the spatiotemporal divergence of viromes sequenced, both in line with the Greek population (500 samples in the same time span of the pandemic) and as an isolated prospective cohort (Figures 1A,B).

### Within-host non-synonymous mutations in serial samples

**FIGURE 2:**
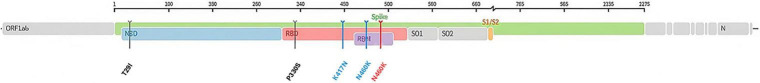
Aggregate figure of within-host mutations in Spike protein (*n* = 3 patients). Alt text: within-host mutations in Spike protein. The graphic was generated from https://covdb.stanford.edu/sierra/sars2/by-patterns/

We studied the eight patients with ≥ 1 follow-up sample and sought to identify within-host mutations leading to amino acid substitutions in the Spike protein, detecting five unique mutations in three patients (Figure 2). Patient #3 (HEM) developed the T29I and K417N mutations after 14 days, patient #11 (RD) developed the P330S and F486V after 5 days and patient #25 (HEM) the N460K mutation after 14 days. T29I mutation is located in the N terminal domain (NTD), whereas the remaining mutations (K417N, P330S, F486V, N460K) in the receptor-binding domain (RBD) of the Spike protein. F486V and N460K are located in the receptor-binding motif (RBM), the main functional part of RBD. In two patients (patient #6 and #11), we identified the loss of K417N and G257D non-synonymous mutations in the follow-up samples. We did not detect any within-host deletions in spike protein. Regarding other viral genome areas, we detected within-host non-synonymous mutations in the follow-up samples of patient #9 (ORF9b:R13H) and patient #25 (M:I49T, ORF1a:T1543A, ORF1b:A1261V, ORF3a:E19Q).

### Intrahost single nucleotide variants (iSNVs) accumulation according to underlying disease

**FIGURE 3:**
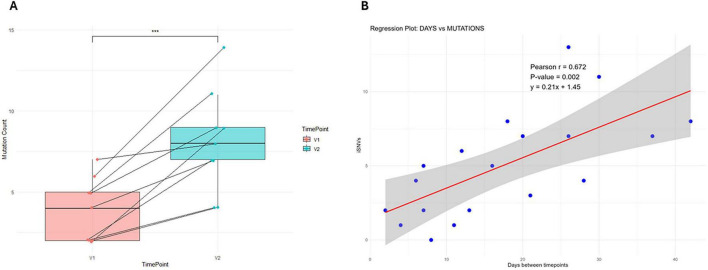
**(A)** Intrahost single nucleotide variants (iSNVs) accumulation between two timepoints (*n* = 8). **(B)** Association between the number of new mutations and duration of symptoms. Alt text: Intrahost accumulation of iSNVs with time. ***p < 0.001.

From the 31 samples included in the genomic analysis, 19 samples with high-quality genomic coverage, including 16 longitudinal samples from 8 patients with two consecutive timepoints, were used to assess the intrahost evolution of the virus. A positive correlation (Pearson’s *r* = 0.67) was found between the number of iSNVs and the duration of infection after symptom onset (average substitution rate per site per day: 7 × 10^–6^). The comparative analysis of the 16 longitudinal samples revealed a statistically significant (*p* < 0.001) accumulation of iSNVs between timepoints (Figures 3A,B).

**FIGURE 4:**
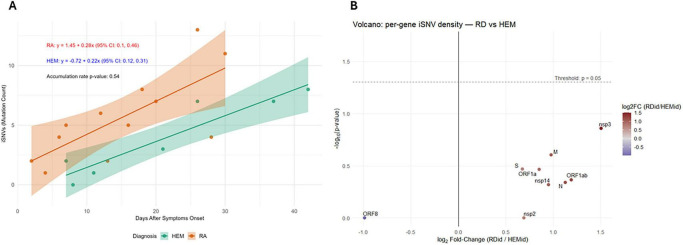
**(A)** New iSNVs between two timepoints per diagnosis. **(B)** Volcano plot of the per gene iSNV density between RD and HEM cases (Wilcoxon rank-sum test was used). Alt text: iSNVs according to diagnosis.

We then grouped the patients into those with RD and HEM diseases, to investigate whether the viral substitution rate differed between these groups. While both groups exhibited an accumulation of iSNVs over time, the mutation rates did not differ significantly (RD: 9.3 × 10^–6^ ± 6 × 10^–6^, HEM: 7.3 × 10^–6^ ± 3 × 10^–6^, *p* = 0.54) (Figure 4A).

We also estimated the normalized iSNV density (substitutions per base pair) for each viral gene (Supplementary Table 3) and we found a diversity in the density between genes, with some not presenting iSNVs (ORF10, ORF7b, ORF6, envelope), while the highest prevalences were observed in nucleocapsid (N, 0.0111 subs/bp), membrane (M, 0.013 subs/bp) and spike genes (S, 0.017 subs/bp). While RD patients showed a slight tendency toward higher iSNV densities compared to HEM, the per-cohort analysis did not yield statistically significant differences (Figure 4B).

## Discussion

We sought to describe the natural course of COVID-19 and the patterns of viral genomic evolution in severely immunocompromised patients. Our main findings include: (i) significant morbidity and mortality; (ii) the rarity of mutations in the protease and polymerase genes, in contrast to the more frequent emergence of mutations in the spike gene; (iii) within-host emergence of spike mutations, particularly in the RBD and its functionally active domain, RBM; (iv) a positive correlation between symptoms duration and the number of accumulated iSNVs; (v) comparable rates of iSNV accumulation between HEM and RD patients, and (vi) variation in mutational potential between viral genes.

B-cell depleted patients due to anti-CD20 mAb therapy can display a markedly altered clinical course of COVID-19. Previous studies have shown that impaired humoral immunity can result in prolonged viral shedding, increased severity and elevated mortality risk (22, 23). Following the rollout of SARS-CoV-2 vaccines, B-cell depletion has been associated with diminished humoral responses, reducing vaccine effectiveness in preventing both infection and severe disease (5). While T-cell responses post-vaccination have not yet been firmly linked to clinical protection (24, 25), in the Omicron-driven era of hybrid immunity, rituximab-treated patients appear less likely to experience severe disease, despite the still increased risk of breakthrough infections (7).

A distinctive aspect of COVID-19 in B-cell depleted patients is the propensity for persistent or relapsing infections, driven by immune evasion from SARS-CoV-2 variants (9). In such hosts, prolonged infection allows the virus to undergo continued replication under partial immune pressure, potentially fostering the emergence of mutations that confer enhanced transmissibility, immune escape or resistance to antiviral therapy (26). The unique environment of a persistently infected host—characterized by suboptimal immune defense and repeated therapeutic interventions—may promote the selection of mutations that enhance viral fitness (27) since, unlike acute infections, this setting provides a prolonged window for such variants to become predominant (28).

A striking finding of our study is that RD patients demonstrated similarly high rates of iSNV accumulation over time compared to HEM patients. This suggests that B-cell depleted RD patients may similarly serve as permissive hosts for SARS-CoV-2 evolution. The analysis revealed a positive correlation between symptoms duration and the number of accumulated iSNVs, consistent with prior studies of prolonged infection in immunocompromised individuals. Notably, RD patients showed a similar burden of iSNV accumulation to HEM patients, despite having different underlying conditions and treatment profiles. While the observed difference in accumulation rates was not statistically significant, the comparable trends are noteworthy and may reflect similar levels of immune impairment—especially in the setting of long-term B-cell depletion through anti-CD20 monoclonal antibodies widely used in rheumatologic practice.

Persistent SARS-CoV-2 infection in immunocompromised individuals has been recognized as a driver of intrahost viral evolution, leading to the emergence of immune escape mutations or antiviral resistance. Our findings extend this concern to RD patients, highlighting that their impaired humoral immunity may allow for similar evolutionary dynamics as seen in hematologic patients. While previous work has largely focused on hematologic or transplant populations, studies specifically examining viral evolution in RD patients remain limited (13, 29) and do not compare the evolutionary process according to underlying disease. Our data contributes to filling this gap by demonstrating that RD patients, particularly those with prolonged infections, can exhibit similar viral evolutionary patterns. This supports that B-cell depletion, regardless of underlying diagnosis, is a key driver of viral persistence and within-host evolution.

Clinical implications that arise are the acknowledgment of risk for persistent infection and viral evolution that should inform the timing and frequency of B-cell depleting therapies, as well as that the prolonged viral shedding could lead to continued infectivity and nosocomial transmission. Furthermore, public health strategies should prioritize these patients for early antiviral therapy as they may act as reservoirs for variant emergence and emphasize the need for vaccination prior to B-cell depletion, when possible. Future studies with larger sample sizes are needed to clarify the role of specific immunosuppressive regimens and immune phenotypes in shaping viral evolution in this population.

The SARS-CoV-2 genes with the highest within-host mutation rate, were those coding for accessory (ORF8) and structural proteins (N, M and S). ORF8 plays an important role in immunity against SARS-CoV-2 via interaction with type I interferon signaling downregulation of class I MHC expression (30, 31) and has shown the highest nonsynonymous over synonymous divergence that any other SARS-CoV-2 gene (32). Regarding the structural genes, Jaroszewski et al have described the higher-than-expected mutation rate of nucleocapsid (N) gene (30). The nucleocapsid protein is also implicated in cell-mediated immunity against SARS-CoV-2, since robust nucleocapsid-specific T-cell responses are necessary during early infection (33). In a study early in the pandemic, Li et al estimated the highest iSNV prevalence in ORF8 gene (1.02 iSNVs/kb) followed by the N gene (0.90 iSNVs/kb) (34). Among all genes, we observed the highest mutation incidence in the Spike gene (17 iSNVs/kb). Other studies have reported a higher normalized incidence in other structural and accessory genes (34, 35). These studies, however, included pediatric and general population and not severely immunocompromised hosts frequently presenting with relapsing infection. Nevertheless, even in the pediatric study, the spike gene had the highest proportion of non-synonymous iSNVs (35). Scherer et al have shown the potential of accumulation of mutations in the spike gene resulting in immune escape in immunocompromised hosts, especially under the selective pressure of mAbs against Spike protein (13). Although none of our patients were treated with such antiviral mAbs, 5 of them (4 included in the final analysis) were treated with intravenous immunoglobulin (IVIG). Recent IVIG preparations contain anti-spike neutralizing antibodies (36) that could have exerted selective pressure for mutations in the spike gene. Unfortunately, the small proportion of IVIG-treated patients prevented us from further analyses. Finally, although the iSNV density comparison between cohorts showed no significant differences, it is important to note that any apparent trends may be influenced by the distinct viral strains circulating within each group and by patient-specific factors.

Among the eight patients with follow-up samples, we identified 5 additional non-synonymous mutations in the spike protein in three of them. T29I is not known to confer immune escape or decreased susceptibility to mAbs. K417N mutation is located in the RBD of the spike protein, is universally detected from early 2022 and confers reduced susceptibility to mAbs. P330S is also located in the RBD domain and at the time of the study it had been detected in only 6 samples in Greece. P330S was detected in a lymphoma patient with chronic SARS-CoV-2 infection, 93 days after diagnosis and after several remdesivir and convalescent plasma courses (37). F486V and N460K are located in the RBM, so their association with immune escape is not unexpected (38).

We believe that our study has significant strengths. These include its prospective design, the patient-level data availability, the homogenous B-cell depleted population, the laboratory-confirmed B-cell depletion, the collection of follow-up samples for within-host viral evolution assessment and the conduction of the study in a specific period during the Omicron predominance. Another important strength of our study is that it is one of the few comparing the iSNV accumulation rate between rheumatic patients and those with hematological malignancies. Our findings further support the evidence that SARS-CoV-2 intrahost evolution is mainly driven by B-cell depletion and not by the underlying disease. Moreover, our strictly tailored iSNV analysis confers a robust method to describe the intra-host evolution dynamics and the diversity of mutation emergence in individuals compared to conventional mutation analyses, providing a powerful tool of detecting mutations with minor frequency but clinical importance as immune escapees. Stringent quality control analysis reduced false positive results and the sufficient number of paired sequenced samples with adequate patient-level data provided our iSNV analysis with further solidity.

Our study does not come without limitations. Among them are the small cohort, the exclusion of samples due to low sampling or sequencing quality and the small number of participants with follow-up samples included in the iSNVs analysis, preventing temporally expanding correlations. Due to the small sample size, we could not perform subanalyses according to gender, age or vaccination status. Some specific disadvantages of iSNV analysis should be noted: (a) overestimation of intrahost diversity due to systematic errors and (b) larger-scale structural variants and small indels in the iSNVs not studied through this analysis. Finally, a drawback is the relatively short interval between the collection of the paired samples. Even within this interval, however, we were able to describe the short-term mutational process of SARS-CoV-2. Finally, since this was a cohort study, the RD and HEM subgroups were not matched, therefore we cannot preclude the existence of confounders that could impact on our results.

In conclusion, B-cell depletion irrespective of the underlying disease, correlates with an increased risk for the emergence of mutations especially in the Spike gene, with no difference in the mutations accumulation rate between patients with rheumatic diseases and those with hematological malignancies.

## Data Availability

The datasets presented in this study can be found in online repositories. The names of the repository/repositories and accession number(s) can be found below: https://www.ncbi.nlm.nih.gov/bioproject/?term=PRJNA1233228, PRJNA1233228.

## Footnotes

1
https://www.bioinformatics.babraham.ac.uk/projects/fastqc/

2
https://github.com/OpenGene/fastp

3
https://clades.nextstrain.org

4
https://clades.nextstrain.org

5
https://docs.nextstrain.org/projects/augur/en/stable/index.html
