# Supplementary material for: Intrahost viral evolution of SARS-CoV-2 infections in rheumatic versus hematological patients with severe iatrogenic immunosuppression
Source: Front Med (Lausanne). 2026 Apr 30;13:1715096. doi: 10.3389/fmed.2026.1715096 (PMC13171318; doi:10.3389/fmed.2026.1715096)

Supplementary material

Human cell isolation from peripheral blood - Flow cytometry

Peripheral blood (12 ml) was obtained from immunocompromised patients that are at risk for increased mortality or protracted/relapsing course of COVID-19. Human peripheral blood mononuclear cells (PBMCs) were enriched by density gradient centrifugation of peripheral blood through a Lymphosep (Biowest, Cat. No. L0560-500) gradient. Shortly, blood was diluted 1:1 with phosphate-buffered saline (PBS) and layered over Lymphosep solution. Falcon tubes were centrifuged at 500g for 30 min with no brake at room temperature. PBMCs were collected, and cells were washed with PBS.

For analysis of immune cell subsets, single-cell suspensions from fresh human PBMCs were stained with conjugated antibodies against: CD4 (1:100, 317444, Biolegend), CD8 (1:100, 301032, Biolegend), CD19 (1:70, 302206, Biolegend). Cells were sorted on a FACSAria III (BD Biosciences) using the BD FACSDiva v8.0.1 software (BD Biosciences). Analysis was performed with FlowJo software. Key resources table including details on the antibodies are shown in Suppl. Table 1.

Suppl. Table 1: Description of the antibodies used in the flow cytometry

Target	Fluorochrome	Clone	Vendor / Source	Cat. / Identifier	Application	Dilution
CD4	BV510	OKT4	Biolegend	317444	FC	1:100
CD8	PerCP-Cy5.5	RPA-T8	Biolegend	301032	FC	1:100
CD19	FITC	HIB19	Biolegend	302206	FC	1:70

Suppl. Table 2: Patients' detailed characteristics

#	Sex (M/F)	Age	Diagnosis	IS therapy	History of prior COVID-19	N of previous vaccine doses	Week of COVID-19 diagnosis[†]	Disease severity at presentation	Clinical relapse	COVID-19 Outcome	N of collected samples	N of samples in the final analysis	Day of sampling after symptoms onset[*]
1	F	74	Retroperitoneal fibrosis	Rituximab, GC	Yes	4	2	Inpatient, not severe	Yes	Discharge	2	0	D17, D25
2	M	62	CLL	Obinutuzumab	Yes	0	3	Inpatient, severe	Yes	Death	1	1	**D10**
3	F	83	CLL	Rituximab, venetoclax	Yes	3	3	Inpatient, not severe	Yes	Discharge	2	2	**D6, D20**
4	F	84	AAV	Rituximab, GC	No	3	3	Inpatient, not severe	No	Discharge	1	1	**D6**
5	M	70	DLBCL	Rituximab, lenalidomide	No	3	4	Inpatient, not severe	No	Discharge	2	1	**D4**, D28
6	F	72	Follicular lymphoma	R-CHOP, GC	Yes	3	8	Inpatient, not severe	Yes	Discharge	3	2	**D7, D18**
7	M	78	RA	Rituximab, methotrexate, GC	Yes	4	9	Inpatient, not severe	Yes	Discharge	1	1	**D8**
8	M	85	DLBCL	Rituximab, cyclophosphamide, GC	No	3	9	Inpatient, not severe	No	Death	1	1	**D15**
9	M	49	Marginal zone lymphoma	Obinutuzumab, GC	Yes	0	7	Inpatient, not severe	Yes	Discharge	3	3	**D12, D26, D39**
10	M	40	CNS vasculitis	GC	Yes	0	10	Inpatient, not severe	Yes	Discharge	2	2	**D7, D26**
11	F	73	AAV	Rituximab, GC	No	2	12	Outpatient	No	Discharge	2	2	**D37, D42**
12	F	22	SLE	Rituximab, cyclophosphamide	Yes	3	12	Inpatient, not severe	Yes	Discharge	1	0	D20
13	M	77	Non Hodgkin's lymphoma	Rituximab	No	4	17	Inpatient, not severe	No	Death	2	2	**D13, 28**

14	F	70	Multiple myeloma	Lenalidomide	Yes	2	21	Inpatient, severe	Yes	Death	1	0	D1
15	M	63	Mantle cell lymphoma	Rituximab, CHOP	Yes	3	16	Inpatient, not severe	Yes	Discharge	1	0	D27
16	M	72	DLBCL	Rituximab, cyclophosphamide	Yes	0	21	Inpatient, severe	Yes	Death	2	1	**D2**
17	F	24	GPA	Rituximab, cyclophosphamide, GC	Yes	3	20	Inpatient, not severe	Yes	Discharge	1	1	**D9**
18	M	50	Hodgkin's lymphoma	Rituximab, lenalidomide, tafasitamab, GC	Yes	3	25	Inpatient, not severe	Yes	Discharge	1	0	D15
19	M	52	SLE	Rituximab, MMF, GC, HCQ	Yes	4	30	Outpatient	Yes	Discharge	1	1	**D8**
20	M	68	AML	Fludarabine, busulfan	No	2	30	Outpatient	No	Discharge	1	1	**D30**
21	F	26	SLE	Rituximab, HCQ, GC	Yes	3	30	Outpatient	No	Discharge	1	1	**D6**
22	F	75	AAV	Rituximab	Yes	3	37	Inpatient, not severe	Yes	Discharge	2	1	D29, **D35**
23	F	76	Dermatomyositis-RA	Rituximab	Yes	4	42	Inpatient, not severe	Yes	Discharge	4	1	D180, D185, D206, **D216**
24	M	78	RA-ILD	Rituximab	No	5	43	Inpatient, not severe	No	Discharge	2	1	**D11**, D19
25	F	61	DLBCL	Obinutuzumab, polatuzumab, tafasitamab	Yes	1	46	Inpatient, not severe	Yes	Death	3	3	**D16, D30, D60**
26	M	83	AAV	Rituximab	No	3	49	Inpatient, severe	No	Discharge	2	2	**D8, D21**

†Week number starting 03.01.2022

**day of collection of the samples included in the final analysis are shown in bold*

AAV: antineutrophil cytoplasmic antibodies-associated vasculitis; AML: acute myeloid leukemia; CLL: chronic lymphocytic leukemia; CNS: central nervous system; COVID-19: coronavirus disease 2019; D: day; DLBCL: diffuse large B-cell lymphoma; F: female; GC: glucocorticoids; GPA: granulomatosis with polyangiitis; HCQ: hydroxychloroquine; ILD: interstitial lung disease; IS:

immunosuppressive; M: male; MMF: mycophenolate mofetil; N: number; R-CHOP: rituximab, cyclophosphamide, doxorubicin, vincristine, prednisone; RA: rheumatoid arthritis; SLE: systemic lupus erythematosus

Suppl. Table 3 Mutations per base per gene (shown in increasing order)

Gene	size in base pairs	mutations in samples	Mutations per base pair
**ORF10**	117	0	0
**ORF7b**	132	0	0
**ORF6**	186	0	0
**E**	228	0	0
**ORF3a**	828	0	0
**nsp6**	870	0	0
**nsp9**	339	0	0
**nsp10**	417	0	0
**nsp11**	39	0	0
**nsp12**	2796	1	0.000358
**nsp4**	1500	2	0.001333
**nsp1**	540	1	0.001852
**nsp15**	1038	2	0.001927
**ORF1ab**	21290	46	0.002161
**ORF1a**	13218	30	0.00227
**nsp3**	5835	14	0.002399
**nsp13**	1803	5	0.002773
**nsp14**	1581	5	0.003163
**nsp5**	918	3	0.003268
**nsp16**	894	3	0.003356
**nsp8**	594	2	0.003367
**nsp2**	1914	7	0.003657
**nsp7**	249	1	0.004016
**ORF7a**	366	2	0.005464
**ORF8**	366	4	0.010929
**N**	1260	14	0.011111
**M**	669	9	0.013453
**S**	3822	66	0.017268

ORF: open reading frame, E: envelope, nsp: non structural protein, N: nucleocapsid, M: membrane, S: spike

Suppl. Figure 1: B- and T-cell flow cytometry analysis. Flow cytometry analysis of immune cells following isolation from peripheral blood of immunocompromised patients at risk for increased mortality or protracted/relapsing course of COVID-19 and extracellular staining (n=21). B cells are characterized as $CD19^+$ cells. T cells are characterized as $CD4^+$ T cells and $CD8^+$ T cells. Frequencies of % of total PBMCs are depicted.

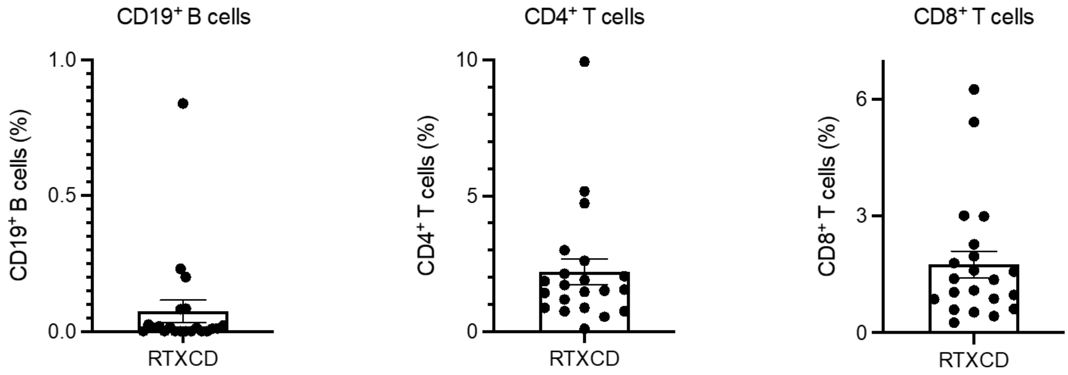

Suppl. Figure 2. Structural representation of the SARS-CoV-2 spike protein was generated using the prefusion spike structure (PDB ID: 6XR8). The spike trimer is shown in ribbon representation, with the receptor-binding domain (RBD) displayed as a semi-transparent surface. Selected monoclonal antibody epitopes are color-coded as follows: Sotrovimab (SOT) in cyan, Tixagevimab (TIX) in green, Cilgavimab (CIL) in yellow, and Bebtelovimab (BEB) in red. Amino-acid changes observed more than 100 times in the GISAID database, indicating epidemiological relevance, are shown in blue. Amino-acid changes occurring at positions known to be associated with phenotypic effects, such as altered host-cell receptor binding or antigenicity, are shown in orange (2A: HEM samples, 2B: RD samples).

2A.

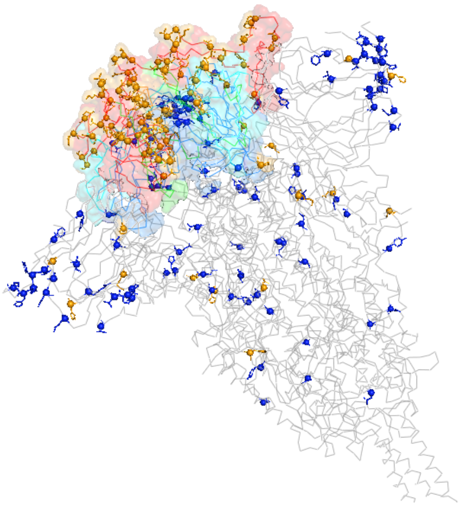

2B.

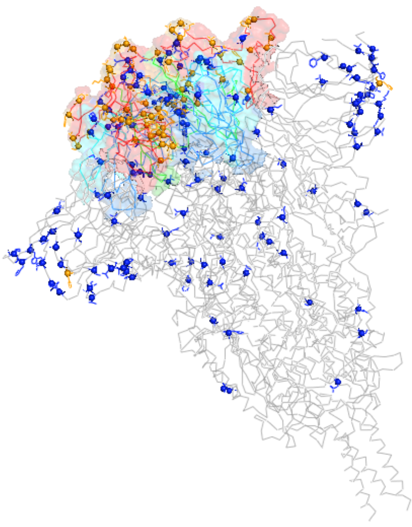

Supplement: Supplementary file 1 [file Data_Sheet_1.pdf]
